# The effect of bone marrow aspiration strategy on the yield and quality of human mesenchymal stem cells

**DOI:** 10.3109/17453670903278241

**Published:** 2009-10-01

**Authors:** Eelco M Fennema, Auke J S Renard, Anouk Leusink, Clemens A van Blitterswijk, Jan de Boer

**Affiliations:** ^1^Institute of Biomedical Technology, University of Twentethe Netherlands; ^2^Department of Orthopaedic Surgery, Medisch Spectrum Twente HospitalEnschedethe Netherlands

## Abstract

**Introduction** Large inter-donor differences exist in human mesenchymal stem cell (hMSC) yield and the response of these cells to osteogenic stimuli. The source of these differences may be clinical differences in stem cell characteristics between individuals or the aspiration procedure itself.

**Methods** From a total of 23 donors, we aimed to take 2 consecutive 10-mL aspirates from the same site in 17 donors and in 6 donors we aimed to take a 5-mL and a 20-mL aspirate. The aspiration was stopped either when the syringe was full or when no more bone marrow came through. Mononuclear cell yield (MNC), MSC yield, and differentiation capacity were analyzed for intra-donor and inter-donor variation. We analyzed the effect of the dilution with peripheral blood by drawing 20 mL at once.

**Results** There was a high correlation between the first and second aspiration volumes, and aspirates with a volume of less than 8 mL showed a large variation in cellular yield. The second 10-mL aspirate, and also 20-mL aspirates, contained a lower concentration of nucleated cells and yielded lower numbers of mesenchymal stem cells. No effect of the aspiration procedure on the biological characteristics of the mesenchymal stem cells was seen.

**Conclusion** We recommend collection volumes of bone marrow aspirates of at least 8 mL to reduce the risk of obtaining aspirates with low cell numbers. From the same site, a second aspiration or an aspirate of > 10 mL can be drawn without any loss of biological quality due to dilution with peripheral blood.

## Introduction

Bone tissue engineering aims to present an alternative for autologous transplantation by using human mesenchymal stem cells (hMSCs), which form functional bone upon implantation (Caplan 1991, Bianco and Robey 2001]). In bone tissue engineering, hMSCs are usually isolated from bone marrow aspirates, induced into the osteogenic lineage in vitro using dexamethasone, and seeded onto 3-dimensional ceramic scaffolds (Caplan 1991). We and others have reported large inter-donor variation in the concentration of nucleated cells and in multi-lineage differentiation potential (Phinney et al. 1999, Siddappa et al. 2007), which hampers standardization of the bone tissue engineering protocol. The success of cell-based bone tissue engineering depends on obtaining a bone marrow aspirate with a high yield and quality of hMSCs. We have found that the MNC and hMSC yield of a given bone marrow aspirate may vary between surgeons (unpublished data). We therefore investigated whether the aspiration process itself might be a source of inter-donor variation.

## Material and methods

### Donors

The 23 donors (7 men) were included after obtaining written informed consent. All donors were undergoing total hip arthroplasty due to coxarthrosis, except for one donor who had a fracture of the femoral neck. The mean age was 66 (22–82) years.

### Isolation and culture of hMSCs

2 consecutive bone marrow aspirates per donor were obtained from the supra-acetabular sulcus. 2 aspirates of 10 mL each were taken from 17 donors using 10-mL syringes, and in addition to this a 5-mL and a 20-mL aspirate were taken from each of 6 other donors (using 10-mL and 20-mL syringes, respectively). The aspiration process was stopped when the syringe was full or when no more bone marrow came through. There was about half a minute between the first and second aspiration. hMSCs were isolated and proliferated as described previously (de Bruijn et al. 1999). hMSC basic medium was composed of hMSC proliferative medium without bFGF; hMSC osteogenic medium was composed of hMSC basic medium supplemented with 10^-8^ M dexamethasone. hMSC cell numbers were determined using a Bürker-Türk counting chamber. Results are given with 95% confidence intervals of the difference between group means (CI). Data were analyzed using t-test with paired comparison for means or Wilcoxon signed-ranks test when the data were skewed (2 observations per donor).

### Proliferation assay

To assess hMSC proliferation, cells from 9 donors were seeded in triplicate in basic medium at 5,000 cells/cm^2^ in T25 tissue culture flasks. They were trypsinized when approaching confluence. Cell numbers were determined and proliferation was expressed as the number of population doublings per day. The means of triplicate determinations were analyzed using t-test with paired comparison for means (18 observations from 9 donors).

### Alkaline phosphatase (ALP) analysis by flow cytometry

hMSCs from 9 donors were seeded at 1,000 cells/cm^2^. Each experiment was performed in triplicate with a negative control (basic medium) and a positive control (osteogenic medium). Expression levels of ALP were analyzed on a FACS Calibur as described previously (de Boer et al. 2004). The means of each triplicate were analyzed using one-way ANOVA followed by Tukey's post hoc test (36 observations from 9 donors).

## Results

### Site-dependent aspiration volume

**Figure 1.**
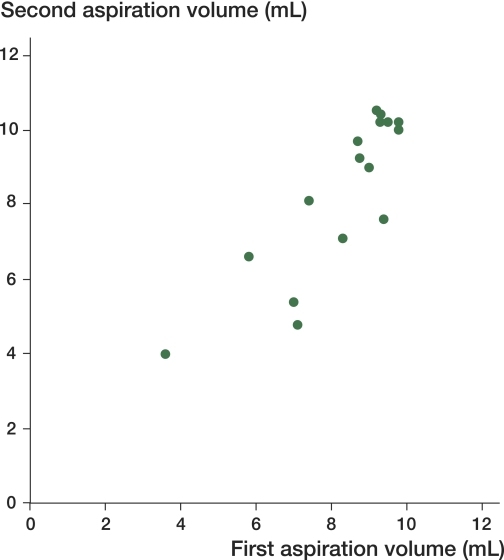
Correlation between the first and second aspiration volumes. Scatter plot of the first and second aspiration volumes. The correlation coefficient (Spearman's rho) is extremely high at 78% (CI: 48–92) but it should be noted that the correlation around 10 mL is artificial because the volume of the syringe was 10 mL.

We tried to collect 2 consecutive 10-mL aspirates of bone marrow from the same site, but noted that sometimes only a small volume could be aspirated. In 15 of the 34 aspirations (17 donors) we could only aspirate up to 8 mL. There was a high correlation between the volumes of the first and second aspirates from each donor (Figure 1).

### The influence of the aspirate volume on the concentration of nucleated cells

**Figure 2.**
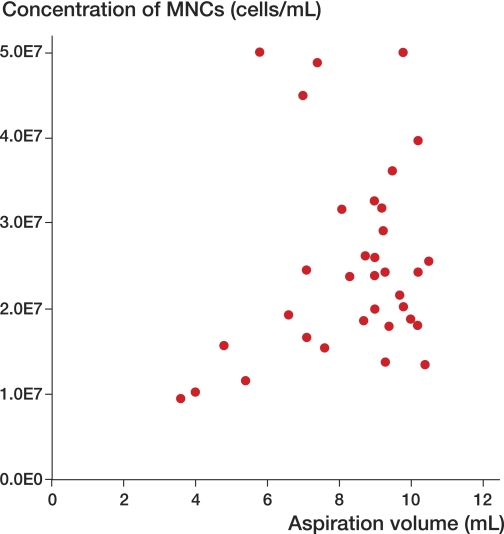
The effect of aspiration volume on the concentration of nucleated cells. Scatter plot of the aspiration volume against the concentration of nucleated cells. With higher aspiration volumes (> 8 mL), the concentration does not appear to increase. The concentrations seem to level off around the mean in the higher aspiration volumes. The mean concentration is 2.3 × 10^7^ (geometric mean). The 4 highest values are not within the 95% confidence interval.

The aspirates that were less than 8 mL yielded a low concentration of MNCs (Figure 2). In contrast, when the aspiration volume was above 8 mL, the concentration of nucleated cells was 2.6 × 10^7^ (SD 0.9 × 10^7^) nucleated cells/mL (23 of 34 aspirations; 2 aspirations per donor).

### Multiple aspirations from the same site is associated with reduced concentration of nucleated cells

**Figure 3.**
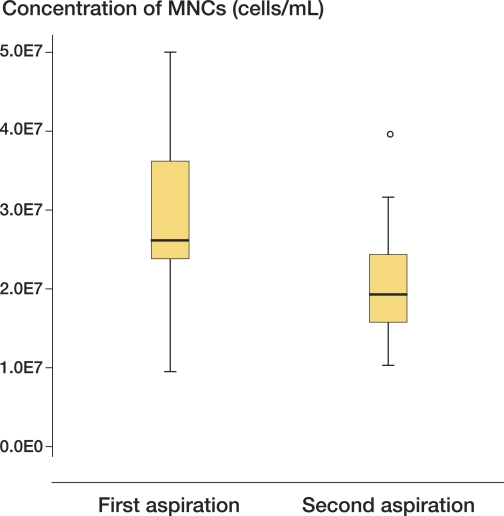
Concentration of nucleated cells per aspiration. Box plots showing the concentration of nucleated cells per mL in the first and the second aspirate with their range, first and third quartile, and mean. The second 10-mL aspirate contained 26% fewer cells on average than the first aspirate. Outlier depicted separately (°).

**Figure 4.**
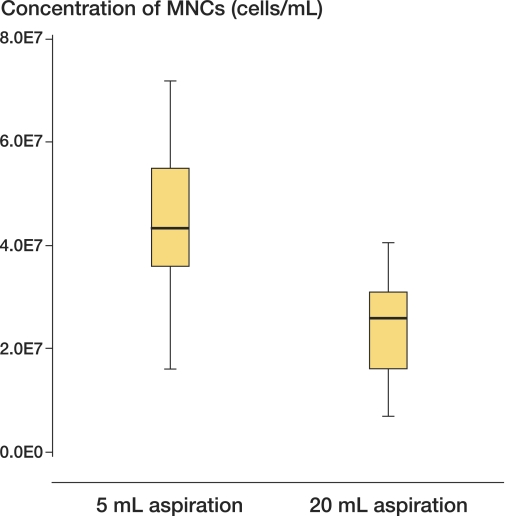
Larger aspiration volumes are associated with reduced concentrations of nucleated cells. Box plots showing the concentration of nucleated cells per mL for the 5-mL and 20-mL aspirates with their range, first and third quartile, and mean. There was a statistically significant decrease in concentration of MNCs in the 20-mL aspirate.

The first aspirate had a higher concentration of MNCs than the second aspirate from the same site: 2.7 × 10^7^ (SD 1.0 × 10^7^) versus 2.0 × 10^7^ (SD 0.6 × 10^7^) MNCs/mL (95%CI: 1.4 × 10^6^ – 1.7 × 10^7^, p = 0.003) (Figure 3). We believe that peripheral blood preferentially refills the drained marrow cavity, due to its lower viscosity than bone marrow. To confirm this, from 6 donors we isolated 5-mL and 20-mL aspirates from the same site consecutively. The 20-mL aspirates were obtained in one effort. The first aspiration of 5 mL resulted in 4.4 × 10^7^ MNCs/mL (SD 1.8 × 10^7^) while the second (20-mL) aspirates yielded a lower concentration of 2.4 × 10^7^ (SD 1.1 × 107) MNCs/mL (95%CI: 1.1 × 10^7^ – 2.9 × 10^7^, p = 0.03) (Figure 4).

### Multiple aspirations from the same site gave reduced yield of hMSCs

**Figure 5.**
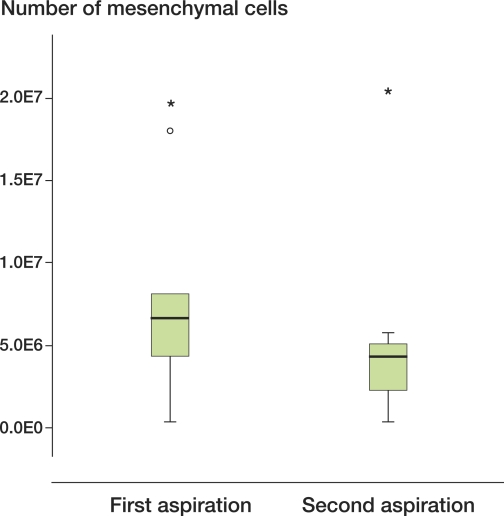
Mesenchymal stem cells from the first and second aspirates. Box plots showing range, first and third quartile, and median for the number of mesenchymal cells derived from the first and second aspirates. Outliers are depicted separately (* and o). After natural logarithmic transformation due to positively skewed data, there was no significant difference between the numbers of mesenchymal cells in the first and second aspirates.

The numbers of hMSCs obtained from the first and second aspirations were grouped according to donor (Figure 5). On average, the second aspiration contained 33% less hMSCs than the first, which was not statistically significant (95%CI: -2.7 × 10^6^ – 8.0 × 10^6^).

### Growth rate of hMSCs isolated from different aspirates

**Figure 6.**
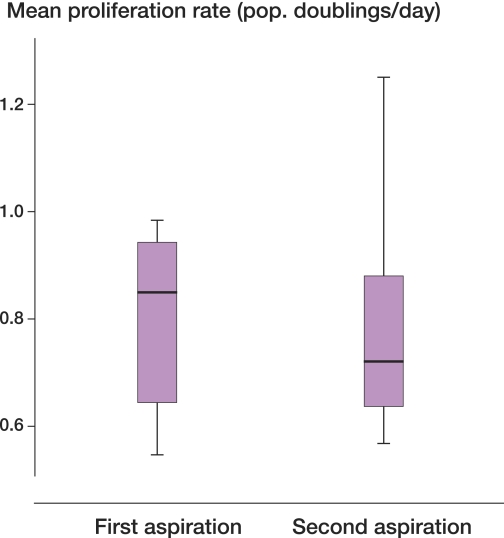
Proliferation rates of the first and second aspiration. Mean proliferation rates for the first and second aspirates. Almost no error was observed and there were only small differences between aspirates. These differences were not significantly different from each other.

The hMSCs obtained from 2 consecutive 10-mL aspirates from 9 donors did not show statistically significant differences in their growth rate in vitro (95%CI: -0.15–0.18) (Figure 6). Moreover, there was no statistically significant correlation between aspiration volume and growth rate of hMSCs (data not shown).

### Osteogenic differentiation of hMSCs isolated from different aspirates

**Figure 7.**
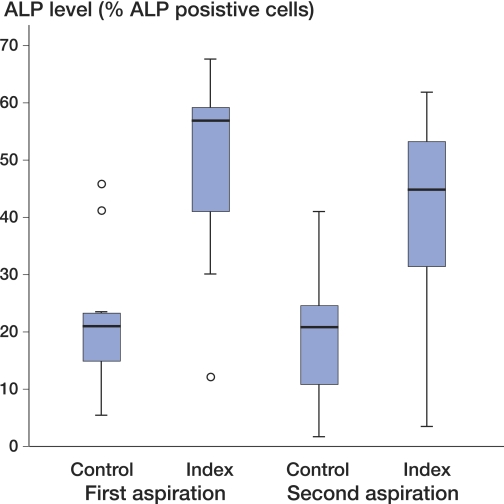
ALP induction per donor. The y-axis shows the percentage of ALP-positive cells gated in flow cytometric analysis. Box plots show the mean ALP levels for triplicate measurements from all donors combined for each modality. The range, first and third quartile, and the median are shown. Outliers are depicted separately (o). One-way ANOVA followed by Tukey's post hoc test showed no significant differences between the ALP levels of the first and second aspirates.

We observed a donor-dependent percentage of ALP-positive cells in untreated and dexamethasone-treated hMSCs (Figure 7). The mean percentage of ALP-positive cells was 21% (2–46) in both untreated groups whereas dexamethasane induction resulted in a mean of 44% (3–68) ALP-positive cells. Both basic and dex-induced ALP expression was lower in the second aspiration; however, no statistically significant differences were observed (95%CI: -0.37–15). We also analyzed the ALP expression per hMSC, which still showed no statistically significant difference in ALP expression (data not shown).

## Discussion

### Each aspiration site has its own volume

Among other variables, the number of hMSCs seeded on the scaffold is indicative of the osteo-inductivity of a graft (Hernigou et al. 2006). Bone marrow is a substance between the sinusoids of spongy bone, which is organized in trabeculae. Trabeculae form crypts (inter-trabecular spaces). Arterioles and venules are only observed in a small proportion of inter-trabecular spaces. This suggests that each arteriole or venule serves several inter-trabecular spaces (Wilkins 1992). The correlation between the lower aspiration volumes for the first and second aspirates implies that an aspiration site has a volume of its own, and this volume reflects the anatomical shape of the underlying intertrabecular space. It could be that the aspiration sites with the lowest volumes are less well interconnected to other intertrabecular sites.

### Donor variation and reduced cell numbers in the second aspirate

When positioning the needle in the bone marrow, and the needle is located near a vessel, peripheral blood will be aspirated more easily than when the needle is located away from it. Since the concentration of MNCs in the peripheral blood is much lower than that in the bone marrow (Ganong 2005), the yield of MNCs in this sample will probably be much lower.

### The influence of peripheral blood on the biological characteristics of stromal cells

We found that the aspiration volume had no effect on the proliferation rate and ALP expression. Studies investigating the effect of acidosis and changes in oxygen concentration on bone formation have generally used a long exposure time (Brandao-Burch et al. 2005, Martin-Rendon et al. 2007). It could be that the difference in circumstances was too little to cause a difference, because once in culture the differences in environmental circumstances are perhaps better equilibrated, when the aspirate has been mixed with culture medium.

In conclusion, we have shown that aspirating twice from the same site reduces cell counts (of MNCs and hMSCs) in the second aspirate without affecting the biological characteristics, and that a low aspiration volume gives an uncertain cellular yield. Increasing the aspiration volume above 8 mL will reduce the cellular concentration of the aspirate to a certain extent, but the biological characteristics remain unaffected by the admixture of peripheral blood. We would therefore like to suggest aspiration of a minimum volume of 8 mL to reduce the chance of obtaining an aspirate with low cell counts, without affecting the biological characteristics of the hMSCs.
